# Comparison of the long-term follow-up and perioperative outcomes of partial nephrectomy and radical nephrectomy for 4 cm to 7 cm renal cell carcinoma: a systematic review and meta-analysis

**DOI:** 10.1186/s12894-019-0480-6

**Published:** 2019-06-07

**Authors:** Yu-Li Jiang, Cheng-Xia Peng, Heng-Zi Wang, Lu-Jie Qian

**Affiliations:** 1 Department of Urology, The Affiliated Hospital of Hang Zhou Normal University, Hangzhou, 310015 China; 2 School of Medicine, Hang Zhou Normal University, Hangzhou, 310016 China

**Keywords:** Renal cell carcinoma, Partial nephrectomy, Radical nephrectomy, Overall survival, Cancer special survival, Recurrence-free survival

## Abstract

**Background:**

The efficacy of partial nephrectomy (PN) for T1b renal cell carcinoma (RCC) is controversial. The oncological outcomes, the change in postoperative renal function and the perioperative complications are unclear.

**Methods:**

We searched PUBMED, EMBASE and the Cochrane Central Register for studies from March 1998 to March 2018 for studies comparing PN to radical nephrectomy (RN) for the treatment of T1b RCC. After data extraction and quality assessment, we used RevMan 5.2 to pool the data. Then, we used Stata 12.0 to perform sensitivity analyses and meta-regression. We used the GRADE profiler to evaluate the evidence according to the GRADE approach.

**Results:**

A total of 16 studies involving 33,117 patients were included in our meta-analysis. No significant difference was found in the 5-year overall survival (OS), 10-year OS, 5-year recurrence-free survival (RFS) and 10-year RFS. The 5-year cancer-special survival (CSS) and 10-year CSS were better in RN compared to PN, respectively, at RR = 1.02, *P* < 0.05 and RR = 1.04, *P* < 0.05. PN was better than RN in the preservation of renal function (WMD = -9.15, 95% CI: − 10.30 to − 7.99, *P* < 0.05). The confidence level grading of the evidence was moderate for 5-year OS, 10-year OS, 5-year CSS, 10-year CSS, 5-year RFS, 10-year RFS, tumor recurrence, decline in eGFR, and postoperative complications.

**Conclusions:**

PN may provide comparable outcomes in terms of RFS & OS, and better renal function preservation although CSS was worse.

**Electronic supplementary material:**

The online version of this article (10.1186/s12894-019-0480-6) contains supplementary material, which is available to authorized users.

## Background

Renal cell carcinoma, also called renal adenocarcinoma, comprises 85% of malignant renal cell carcinomas (RCCs), accounts for 2–3% of all cancer diagnoses and is a common urology cancer [1]. The increased chance of detection of small renal masses has attracted urology surgeons to focus on making and revising operation methods and treatments for T1b renal cell carcinoma. The incidence of diagnosis of RCC has increased steadily by 2.5% (the most common histological variant of RCC is clear-cell carcinoma).

In the last several decades, radical nephrectomy (RN), has been the standard treatment for RCC. Since 1980, Nov et al. reported that partial nephrectomy has been adopted for some selective renal cell carcinoma patients [2]. PN has been accepted as a standard surgical approach, especially in the last decade, for the treatment of patients with a renal mass < 4 cm, with equivalent oncological outcomes and better renal preservation than RN [3]. Initially, PN was performed solely in patients with imperative indications, but it is also being adopted for patients with relative indications. Even though both EAU guidelines and AUA guidelines recommend the use of PN as a standard treatment, evidence supporting this recommendation is insufficient [4, 5]. The present study showed that the oncological long-term outcomes between patients treated with PN and RN were similar, but patients treated with RN had a higher mortality rate than those treated with PN [6–9]. However, there are few studies addressing the surgical indications for the treatment of T1b renal tumors.

The advantages of PN for the treatment of T1b renal tumor are still controversial. Several studies have reported that PN could have a similar effect on the control of cancer as RN. Kim et al. performed a meta-analysis to compare the outcomes of PN and RN in cases of localized renal tumors. They reported that PN had a greater advantage over RN [10]. However, the studies involved patients with T1a renal tumor masses. The study reported cancer-specific mortality and all-cause mortality. In addition, Mir et al. conducted a meta-analysis comparing PN and RN and combined cases with T1b and T2 renal tumor masses. Therefore, we performed a meta-analysis comparing PN and RN for the treatment of patients with T1b RCC.

The aim of this meta-analysis was to compare long-term oncological outcomes in patients with 4 to 7 cm RCC treated with PN and RN.

## Methods

This meta-analysis was performed in accordance with the Preferred Reporting Items for Systemic Reviews and Meta-analysis (PRISMA) statement. We searched PUBMED, EMBASE and the Cochrane Central Register for studies which published in English dating from March 1998 to March 2018. The search terms were renal tumors, kidney cancer, renal cancer, T1b, 4–7 cm, nephron-sparing-surgery or PN, radical nephrectomy, PN and RN, NSS and RN. We also used the combined Boolean operators “AND” or “OR” Title/Abstract. The two investigators (YLJ and HZW) reviewed the results in the case of discrepancies. The inclusion criteria were as follows: (1) comparative PN or NSS and RN for the treatment of 4–7 cm renal tumors and (2) evaluation of at least one oncological result such as RFS, CSS, and OS.

**Fig. 1:**
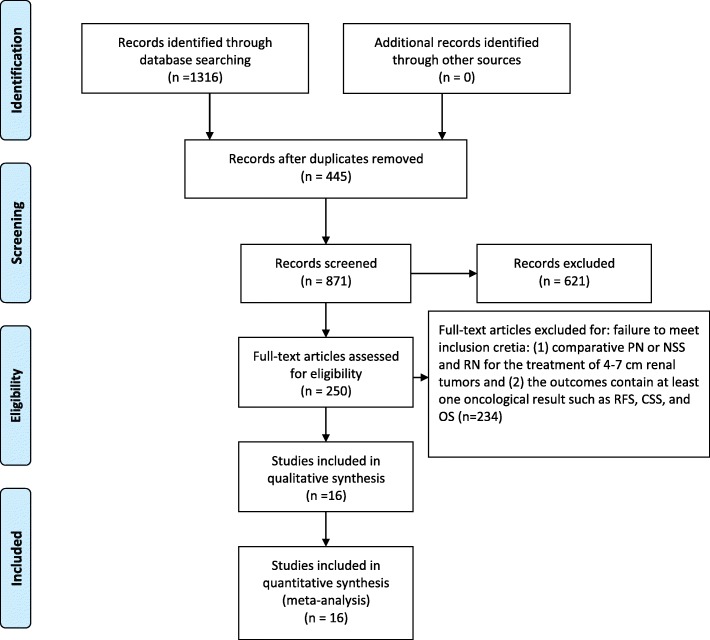
Flow diagram of the process for the selection of relevant studies

The exclusion criteria were as follows: (1) case reports, reviews, and articles without applicable data; (2) studies that included both T1b and T2 or renal tumors larger than 7 cm; and (3) studies that were not comparative in nature. The process of identifying relevant studies is summarized in Fig. 1.

### Statistical analysis

We used Review Manager Version 5.2 software (The Cochrane Collaboration, Oxford, UK) and Stata 12.0 to perform the analysis of the included data. We used the GRADE approach to evaluate the quality of the evidence. We used Cochran’s Q to evaluate the heterogeneity; if the value of Q < 50% or *P* > 0.01, we believed little heterogeneity was present. However, if Q > 50%, *P* < 0.01, evident heterogeneity existed. For quantitative data, we used weight mean difference (WMD) or standard mean difference (SMD) to calculate continuous data. We used OR and 95% confidence interval (CI) to evaluate binary data. We used LnRR instead of RR and SE to normalize the distribution of the data. We used Stata 12.0 to perform funnel plots to evaluate the publication bias among the studies (Additional file 1: S1 File).

### Evidence grading

We used the GRADE profiler to create the evidence grading. Assessment of the underlying quality of the evidence, major statistical results, and grading of the evidence for each outcome made the body of the evidence. The evidence was graded as high, moderate, lower, low or very low quality. The criteria for the evaluation of the evidence included the assessment of risk bias as determined from the results of the Cochran Risk of Bias Tool.

## Results

A total of 1316 studies were obtained from the selected databases. The PRISMA flow diagram of the search process is summarized in Fig. 1. We removed 445 duplicates. From the selected databases, we obtained 144 reports. After screening the titles and abstracts, 89 full texts were excluded for failure to meet inclusion cretia. After detailed processing, 29 reports were excluded for failure to meet inclusion cretia. Finally, sixteen studies were included in our meta-analysis [11–26]. Table 1 summarizes the baseline characteristics and assessments.

**Table 1:** Basic Characteristics of the Included Studies

Study	Study type	Group	Sample Size	Age(y)	Sex F/M	Pathologic tumor size (cm)	Follow-up month(s)
Kim2012	retrospective	PN	18	47.3	5/13	5.0	78.2
		RN	52	57.3	16/36	5.5	66.5
Badalato2011	retrospective	PN	1047	58.4	677/370	4.86	120
		RN	10,209	60.7	4166/6043	5.27	120
Jang2016	retrospective	PN	100	55.3	29/71	4.9	48.1
		RN	100	55.7	29/71	4.9	42.6
Iizuka2012	retrospective	PN	67	57.6	16/51	4.9	31.3
		RN	195	58.1	76/119	5.3	93.0
Roos2011	retrospective	PN	73	60.4	7/66	5.0	55.2
		RN	100	61.1	62/38	5.5	78
Roos2012	retrospective	PN	85	NA	55/56	NA	120
		RN	118	NA	59/87	NA	120
Crepel2010	retrospective	PN	275	60.5	100/175	NA	40.7
		RN	1100	60.5	433/667	NA	46.7
Milonas2013	retrospective	PN	34	62.2	9/15	4.67	168
		RN	317	63.4	153/164	5.25	168
Thompson2009	retrospective	PN	286	NA	90/196	NA	57.6
		RN	873	NA	335/538	NA	57.6
Pignot2014	retrospective	PN	123	57.6	43/80	5.2	39.5
		RN	185	61.6	58/127	5.5	46.9
Antoneli2012	retrospective	PN	198	58.2	64/134	5.0	120
		RN	1426	62.4	524/902	5.7	120
Meskawi2013	retrospective	PN	1526	60.3	493/1033	5.14	60
		RN	6104	60.6	1966/4138	5.15	120
Weight2010	retrospective	PN	212	49	73/139	4.8	49
		RN	298	41	122/176	5.6	41
Simmoons2009	retrospective	PN	35	63.5	9/26	4.6	NA
		RN	75	63.4	36/39	5.3	NA
Robert2006	retrospective	PN	33	68.9	7/26	5.2	34.0
		RN	66	66.9	22/44	5.2	48.5
Patard2004	retrospective	PN	64	NA	NA	5.3	120
		RN	576	NA	NA	5.6	120

*NA* Not Available

### Quality assessment of the included studies

We used the New-Ottawa Scale (NOS) to assess the included nonrandomized studies. We used a 9-point system to evaluate the NOS scores. The study score of 7–9 or above was considered high quality, a score of 4–6 was considered medium quality, and a score of 0–4 or below was considered low quality. Two reviewers (YLJ and CXP) evaluate the quality of the included studies. Table 2 shows the risk of bias of the selected studies.

**Table 2:** Newcastle-Ottawa Scale for risk of bias assessment of the included studies

Study Design		Selection				Comparability	Outcome			Total
		Representativeness of exposed cohort	Selective of nonexposed Cohort	Ascertainment of exposure	Outcome not present at start		Assessment of outcome	Adequate follow-up length	Adequacy of follow-up	
Kim	R	*	*	*	*	**		*	*	8
Badalato	R	*		*	*	**		*	*	7
Jang	R	*	*	*	*	*	*	*	*	8
Iizuka	R	*		*	*	**	*	*	*	8
Roos2011	R	*		*	*	**	*	*	*	8
Roos2012	R	*		*	*		*	*	*	8
Crepel	R	*	*	*	*	**	*	*	*	9
Milonas	R	*		*	*	*	*	*	*	7
Thompson	R	*		*	*	**	*	*	*	8
Pignot	R	*		*	*	**	*	*	*	8
Antoneli	R	*		*	*	**	*	*	*	8
Meskawi	R	*		*	*	**		*	*	7
Weight	R	*	*	*	*	**	*	*	*	9
Simmoons	R	*		*	*	**	*	*	*	8
Robert	R	*	*	*	*	**	*	*	*	9
Patard	R	*		*	*	**		*	*	8

*R* Retrospective study

### 5-year OS and 10-year OS

**Fig. 2:**
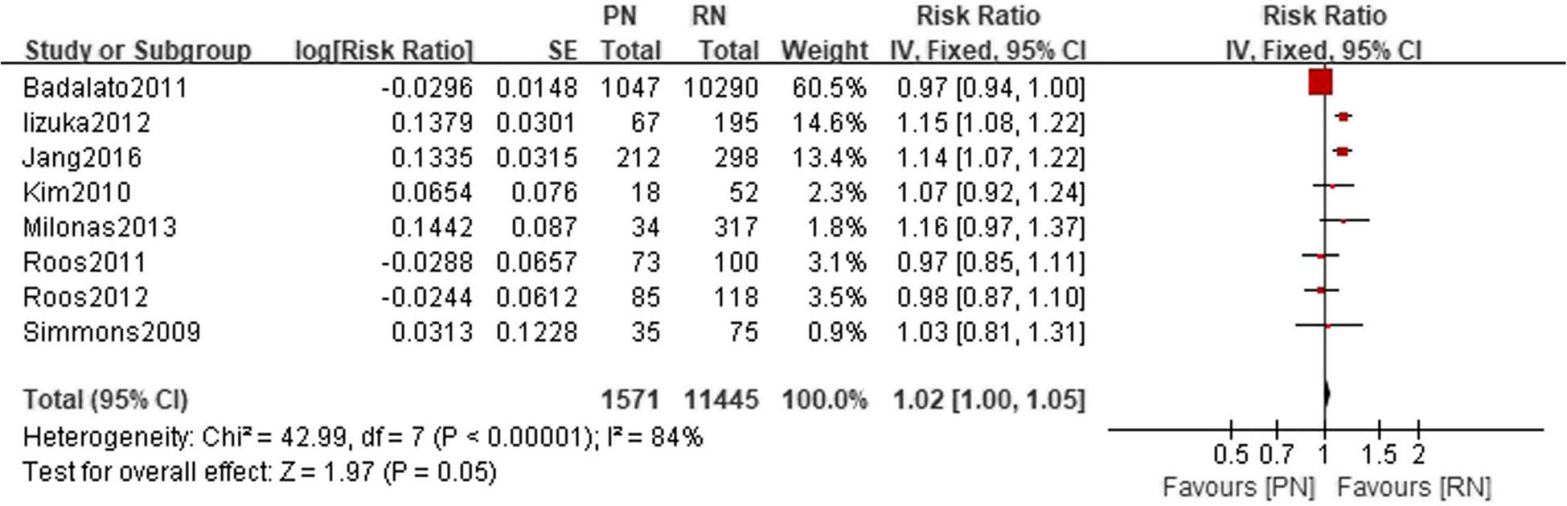
Forest plot for 5-year OS between the PN and RN for T1b tumors

**Fig. 3:**
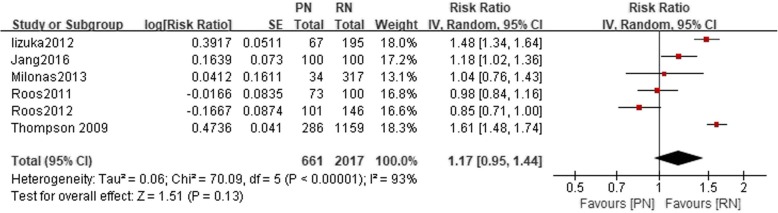
Forest plot for 10-year OS between the PN and RN for T1b tumors

Eight studies reported the 5-year OS outcome. There was no significant difference in the 5-year OS between the PN and RN groups (*n* = 13,016, 1571 patients were in the PN group, 11,445 patients were in the RN group, 95% CI 1.0–1.05; *p* = 0.05, I^2^ = 84%, fixed-effect model, Fig. 2). Six studies reported the 10-year OS in our meta-analysis. No significant statistical difference between the PN and the RN group was noted (*n* = 2678, LnRR: 1.17, 95% CI: 0.91 to 1.10, I^2^ = 85%, fixed-effects model, Fig. 3).

### 5-year CSS and 10-year CSS

**Fig. 4:**
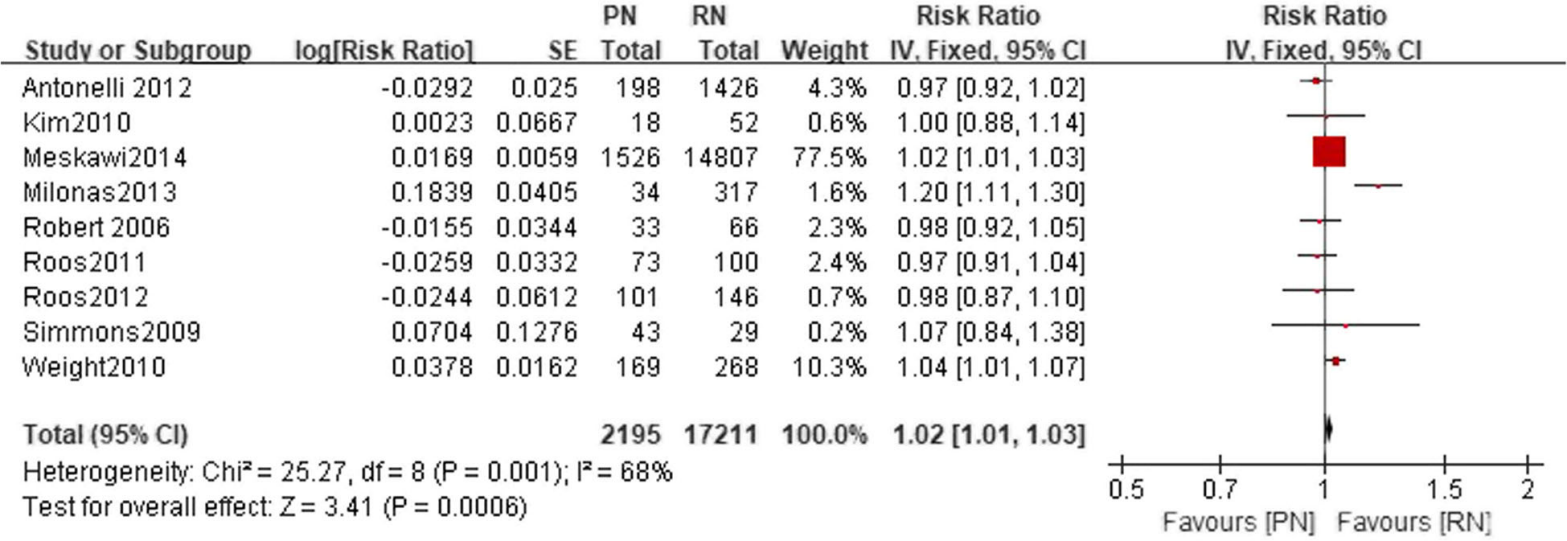
Forest plot for 5-year CSS between the PN and RN for T1b tumors

**Fig. 5:**
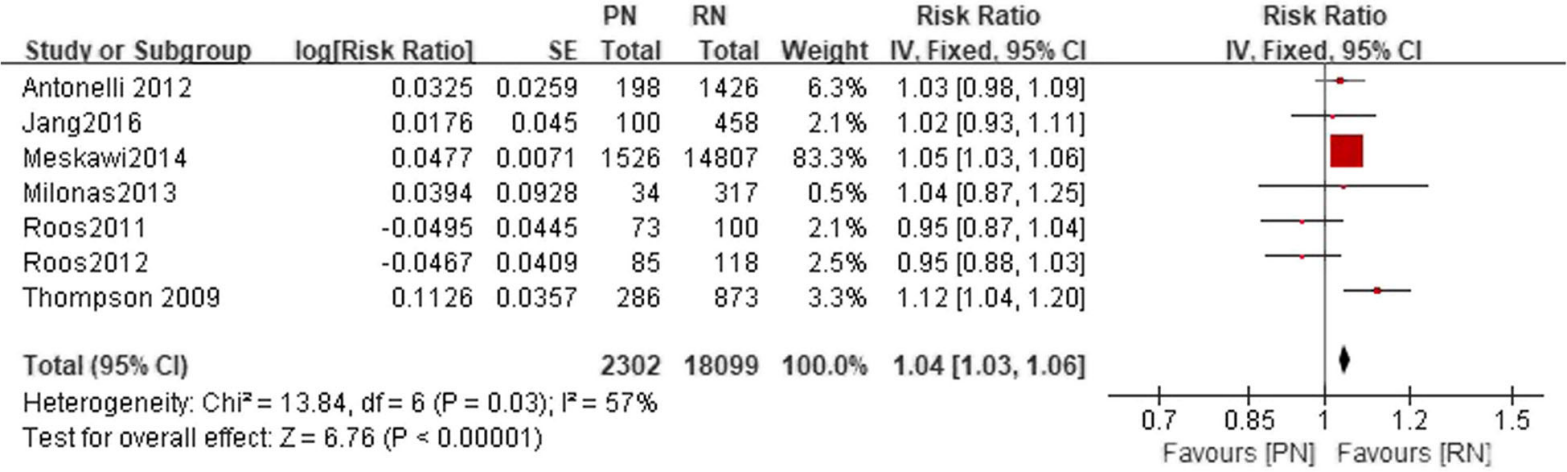
Forest plot for 10-year CSS between the PN and RN for T1b tumors

Nine studies included a 5-year CSS. There was a statistically significant difference between the PN and the RN group (*n* = 19,406, 2195 patients were in the PN group, 17,211 patients were in the RN group, LnRR: 1.02, 95% CI 1.01–1.03, *p* = 0.001, I^2^ = 68%, fixed-effective model, Fig. 4). Seven studies included a 10-year CSS. There was a statistically significant difference between the PN and the RN group (*n* = 20,401, 2302 patients were in the PN group, 18,099 patients were in the RN group, LnRR: 1.04, 95% CI 1.03–1.06, *p* < 0.05, I^2^ = 57%, fixed-effective model, Fig. 5).

### 5- and 10-year RFS

**Fig. 6:**
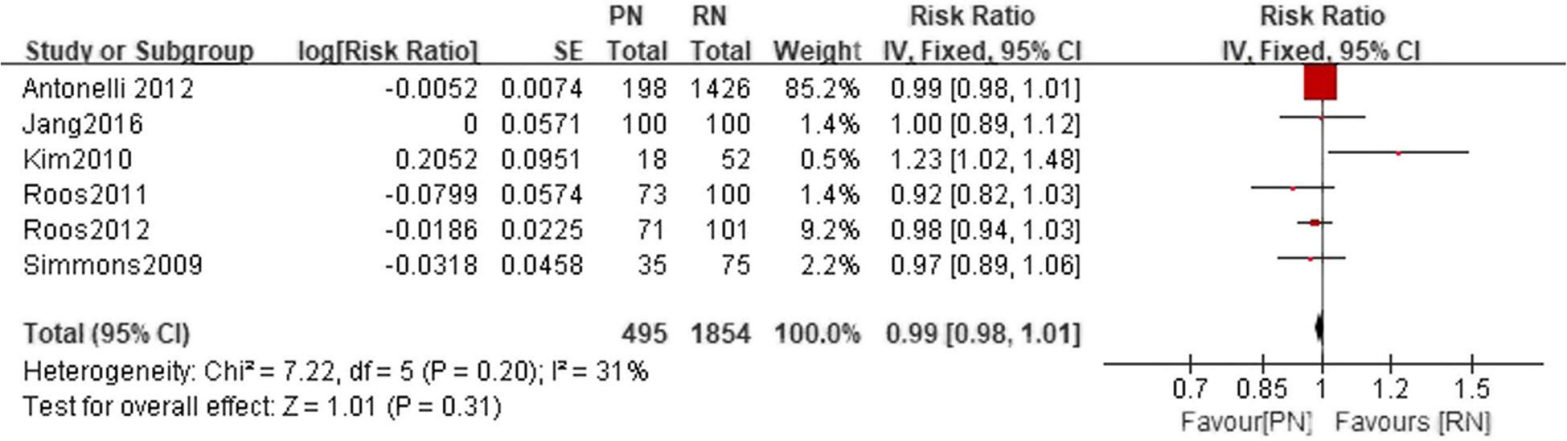
Forest plot for 5-year RFS between the PN and RN for T1b tumors

**Fig. 7:**
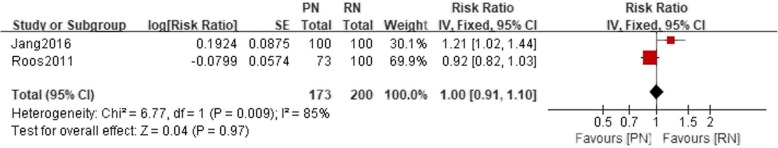
Forest plot for 10-year RFS between the PN and RN for T1b tumors

Six studies reported the 5-year RFS. No significant difference was found between the PN and the RN group (*n* = 2349 patients, 495 patients were included in the PN group, and 1854 patients were included in the RN group, LnRR: 0.99, 95% CI: 0.98 to 1.01, χ^2^ = 7.22, df = 5, *p* = 0.20, I^2^ = 31%, fixed-effects model, Fig. 6). Two studies reported the 10-year RFS. No significant difference was found between the PN and the RN group (*n* = 373 patients, 173 patients were included in the PN group. and 200 patients were included in the RN group, LnRR: 1.00, 95% CI: 0.91 to 1.10, χ^2^ = 6.77, df = 1, *p* = 0.97, I^2^ = 85%, fixed-effects model, Fig. 7).

### Tumor recurrence

**Fig. 8:**
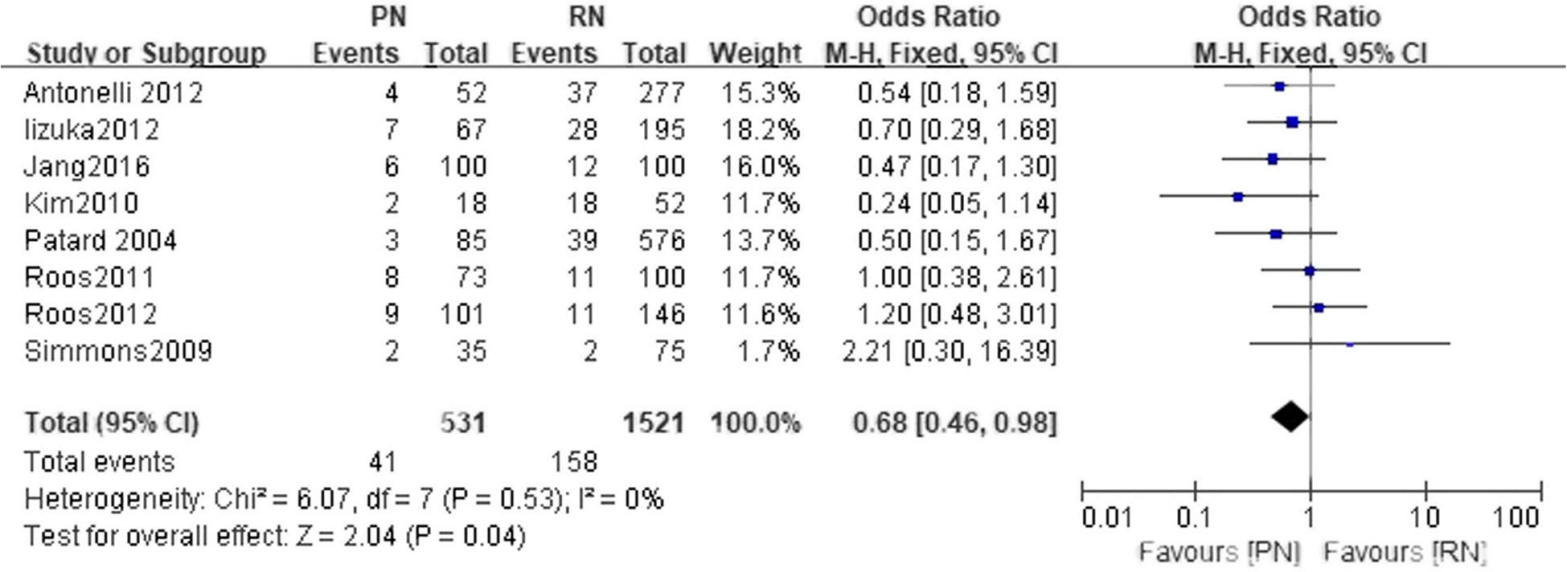
Forest plot for Tumor recurrence between the PN and RN for T1b tumors

Eight studies included in our meta-analysis assessed tumor recurrence. The patients in the PN group had a lower rate of tumor recurrence compared to the RN group (*n* = 2052 patients, 531 patients were in the PN group, 1521 patients were in the RN group, OR: 0.68, 95% CI: 0.40 to 0.98, χ2 = 6.07, df = 7, *p* = 0.04, I^2^ = 0, fixed-effects model, Fig. 8).

### Postoperative complications

**Fig. 9:**
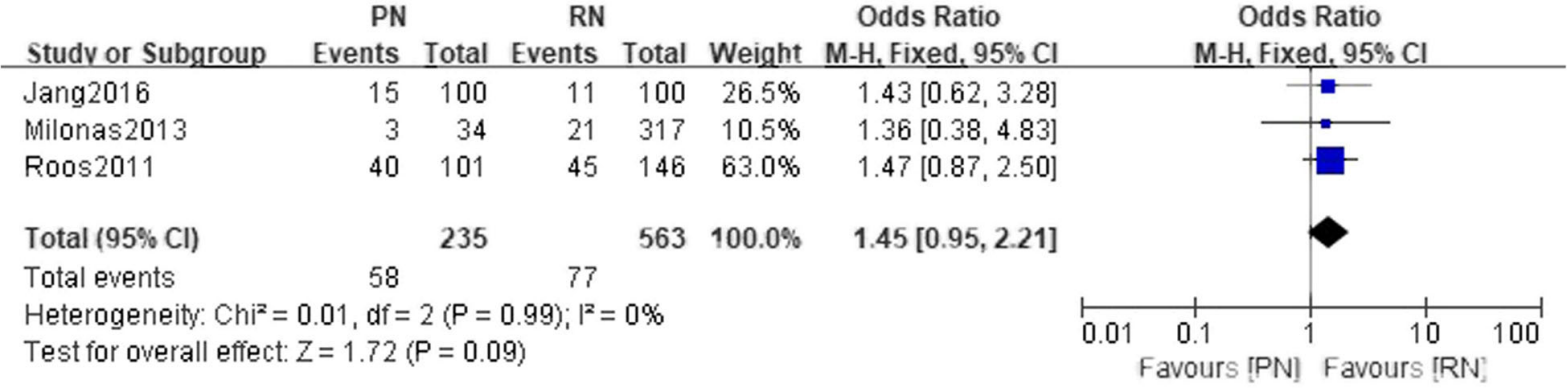
Forest plot for postoperative complications between the PN and RN for T1b tumors

Three studies reported postoperative complications. There was no statistically significant difference between the PN and the RN group (*n* = 798 patients, 235 patients were in the PN group, 563 patients were in the RN group, LnRR = 1.45, 95% CI: 0.95 to 2,21, I^2^ = 0, χ^2^ = 0.01, df = 2, *P* = 0.99, fixed-effect model, Fig. 9).

### Declined eGFR

**Fig. 10:**
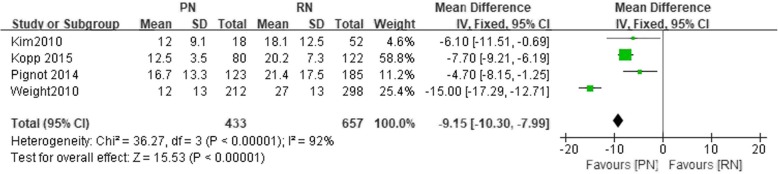
Forest plot for Declined eGFR between the PN and RN for T1b tumors

Four studies reported declined eGFR. There was a significant difference in declined eGFR between the PN and the RN group (*n* = 1090, 433 patients were in the PN group, 657 patients were in the RN group, WMD; − 9.15, 95% CI: − 10.30 to − 7.99, χ^2^ = 36.27, df = 3, *P* < 0.05, I^2^ = 0.92, fixed-effects model, Fig. 10).

### Sensitivity analysis

We sequentially omitted a single study one by one to determine which studies contributed the most to the heterogeneity of the results. In the 5-year and 10-year OS outcomes, we found that the studies performed by Badalato (2011) and Thompson (2009) greatly changed the overall outcome. In the declined eGFR outcome, the study performed by Weight (2009). probably changed the outcome greatly. In the 5-year CSS outcome, no study changed the outcome significantly. (Additional file 2: S2 File).

### Grading assessment

Additional file 3: Figure S3 shows a summary of evidence grading. The evidence was graded as moderate for 5-year OS, 10-year OS, 5-year CSS, 10-year CSS, 5-year RFS, 10-year RFS, tumor recurrence, declined eGFR, and postoperative complications. (Additional file 3: S3 File).

## Discussion

The present systematic review and meta-analysis was performed to compare the long-term follow-up outcomes and perioperative outcomes of partial nephrectomy and radical nephrectomy for the treatment of T1b renal cell carcinomas. The present study was the first meta-analysis to compare long-term oncological outcomes. In addition, the present study provides us with moderate evidence determined by using the GRADE approach to evaluate the effectiveness of PN versus RN for the treatment of T1b renal tumors.

Our meta-analysis demonstrated that patients with T1b RCC presented comparable basic characteristics. Our study compared the long-term follow-up outcomes among patients receiving PN versus RN. These patients shared comparable common baseline characteristics. In our study, 5-year CSS, 5-year RFS and 5-year OS rates were not significantly different for patients undergoing PN versus RN. The indications for PN are exclusively for 4–7 cm RCC. In the present study, no significant differences in 5-year and 10-year OS were found between the PN and the RN groups for the treatment of T1b renal tumors. Zhang et al. found that OS was independent of the type of surgical procedure in the propensity score matching [27].

Our present study demonstrated that the 5-year and 10-year OS were not impacted by PN or RN. In addition, Weight et al. found that patients with T1b RCC had a better OS in the PN group compared to the RN group. Antonelli et al. and Thompson et al. also reported similar outcomes [11, 18]. However, Zhang et al. reported that the PN group had a better OS according to both Cox-regression and Kaplan-Meier analysis (*P* < 0.01). Several studies also reported that PN or RN do not influence the OS. These studies used propensity score matching to eliminate any potential confounding factors. Tumor size, tumor grading and age may influence the OS of the two groups. After propensity score matching, no significant difference was found in the baseline covariates, which lowered the effects of potential confounders in the regression models. In addition, it was found that the OS of T1b RCC following treatment was similar between the PN and the RN groups in cases where patients with underlying conditions were treated with PN. Roos et al. found that the difference in OS was related to selection bias. Additionally, they found that tumor size was an independent factor affecting the OS of patients treated with RN instead of PN. Roos et al. reported that tumor size was related to a reduced OS for patients with RN and not PN. They also demonstrated that the feasibility of PN was dependent upon tumor location [24]. The selection bias present in the included studies with regards to the tumor pathology (clear-cell carcinoma vs non-clear cell carcinoma) may account for the higher heterogeneity of the 10-year OS outcome.

Our study showed that patients receiving RN for the treatment of a T1b renal tumor had a longer 5-year and 10-year CSS compared to those receiving PN. Similarly, Leibovich et al. reported that patients receiving PN had a higher 5-year CSS [8]. However, several articles reported no significant difference in the 5-year CSS rate in patients with T1b RCC treated with PN versus RN [17, 23, 25]. Tumor location, tumor size and selection bias may account for this difference. It is believed that selection bias may have influenced these results. Additionally, these differences may also be due to the cancer subtype and TNM stage. Crepel et al. reported the CSS rate and suggested that PN should have equal consideration [12]. However, Lee et al. found no significant difference between the two types of treatment using Cox analysis. These differences did not persist after adjusting for some preoperative covariates. In their study, the patients were usually younger and had smaller tumors in the PN group than in the RN group. They showed that age, tumor size and T stage may be potential predicting factors to evaluate the CSS [14]. In our study, the patients in the PN presented a smaller tumor size than those in the RN group. This factor may have contributed to the results.

Several studies showed that the CSS was better in the PN than in the RN group. Weight et al. and Thompson et al. found that patients receiving RN had almost a twofold greater mortality than those receiving PN [18]. Milonas et al. found that the estimated 12-year CSS was lower than RN, thus indicating that disease relapse was lower than that in patients receiving RN [18]. Antonelli et al. found that the CSS decreased in the case of patients receiving PN for Fuhrman grade IV RCC (clear cell carcinoma) [11].

Weight et al. reported,a total of 510 patients, that the lack of histological subtyping and tumor size did not have a significant effect on the outcome [19].

Tumor recurrence in patients with T1b renal tumors who received PN was lower than those who underwent RN. Similarly, Milonas et al. and Mitchell et al. reported that PN was associated with a higher risk of tumor relapse compared to RN [16, 18]. A recent study performed by Roos et al. reported that patients with papillary renal carcinoma had a higher tumor recurrence rate than those with clear cell carcinoma [26]. In addition, Robert also found that there was no significant difference in recurrence-free survival and cancer-specific survival in patients with T1b RCC treated by PN versus RN [16]. However, Patard et al. found that RN was associated with a lower rate of local and systemic recurrence compared to PN [17]. This was different from the results shown in our study. Furthermore, they found that altering the type of surgical intervention (PN or RN) did not result in a higher incidence of tumor recurrence. In addition, Leibov et al. reported that no significant difference was found in CSS and distant metastasis rates between PN and RN. They also found that tumor stage was not related to tumor recurrence [8]. This may be associated with the histology of the renal tumor and the clinical stage or the pathology stage. Additionally, these studies had a shorter follow-up period. Furthermore, the surgical expertise of the urologist may have also contributed to the selection bias. Additionally, Patard et al. found that the two surgical approaches may impact tumor recurrence. Tumor recurrence may be linked to the histological tumor subtype and grade. The relationship between tumor recurrence and positive margins is unclear. In addition, it is unclear whether the selection of PN or RN is relevant to pathology of the tumor. Lastly, the clinical stage of an RCC is different from the pathological stage [17].

In our study, for patients with larger T1b renal tumors, patients in PN were comparable with RN for 5-year RFS and 10-year RFS. Robert et al. also found that the tumor diameter was an independent factor related to RFS. They found that the histological subtype, stage, grade, and surgical technique were not significant predictors of recurrence-free survival [16].

This was compared with our study. Lu et al. found that tumor enucleation was associated with a lower morbidity and mortality compared to PN [28].

Several studies also reported that preservation of renal function could lower the risk of cardiovascular complications and increase the OS, indicating that PN has the advantage of preserving renal function [13, 19, 22, 25, 29]. Lane et al. also found that patients with preexisting CKD were at a higher risk of death by increasing the incidence of patients developing chronic renal insufficiency. Therefore, it was unclear whether PN or RN were factors impacting CKD via different routes [30]. Roos et al. also determined via multivariate analysis that tumor size was not an independent predictor and was not associated with CKD [26]. Badalato et al. found that, due to the preservation of renal function, patients receiving PN had a higher health-related quality of life [23]. Simmons also proposed that the main factor restricting the selection of surgical methods was tumor location rather than tumor diameter [25]. In addition, the indications for PN should be broadened to reduce the chance of the development of postoperative CKD. Additionally, Pignot et al. reported that tumor size, receiving PN and age are all factors that could impact renal function loss [22].

In our study, the rate of postoperative complications was comparable between PN group and RN group. Several articles also reported a similar result [11, 14, 15]. However, Mir MC et al. performed a meta-analysis demonstarting that the PN had a higher likelihood of postoperative complications than RN (RR 1.74). They included both clinical T1b and T2 tumors [31]. Van Poppel H et al. reported a similarly results founding that higher urinary fistula and reoperation rate in PN [32]. In addition, Meskawi et al. reported that, after a learning phase, PN would become feasible in urology [15].

Furthermore, uncommon RCCs, for example, papillary RCC, may present metastasis to distant locations. Therefore, for papillary RCC larger than between 4 cm and 7 cm, RN should be selected. The urologist should decide on which surgical approach to take to treat T1b RCC based on tumor location, histology of the tumor, mean Fuhrman grade, and tumor diameter. In addition, the PN group may have a higher mortality rate. Using multivariate analysis, Weight et al. reported that patients receiving RN had a higher risk of death than those receiving PN.

Our study has several limitations. First, the included studies were non-RCTs, which may reduce the quality of the evidence used. Second, our meta-analysis used LnRR instead of LnHR to demonstrate the OS, CSS and RFS. Third, the studies included in suffered from bias, especially selective bias (surgeons use PN or RN according to their personal preference), thus introducing heterogeneity. Additionally, the studies included in this study used different surgical techniques, such as LPN, OPN, a fact which may have increased the overall bias. Furthermore, the tumor histology could, to an extent, have affected the postoperative follow-up outcomes. However, after sensitivity analyses and meta-regression, no explicit causes of heterogeneity have been detected. Further studies should determine whether tumor location, tumor diameter or tumor grading are determining factors in the selection of PN or RN.

## Conclusions

Our meta-analysis showed that PN may provide comparable outcomes in terms of RFS & OS, and better renal function preservation. In addition, RN ensures a better CSS compared to PN. PN had an equal incident of postoperative complications compared to RN.

## Additional files

Additional file 1: **Figure S1.** (a)-1(d). Funnel plots of the study. (a) 5-year css (b)5-year os (c)10-year os(d) Declined eGFR. (ZIP 82 kb)

Additional file 2: **Figure S2.** (a)-2(d) Sensitive analysis of the (a) 5-year css (b)5-year os (c)10-year os(d) Declined eGFR. (ZIP 95 kb)

Additional file 3: **Figure S3.** Summary of evidence grading. (PNG 84 kb)

## Data Availability

All data generated or analyzed during this study are included in this published article.

## Abbreviations

CSS: Cancer-special survival
NOS: New-Ottawa Scale
NSS: Nephron-sparing-surgery
OS: Overall survival
PN: Partial nephrectomy
PRISMA: Preferred Reporting Items for Systemic Reviews and Meta-analysis
RCC: Renal cell carcinoma
RFS: Recurrence-free survival
RN: Radical nephrectomy
SMD: Standard mean difference
WMD: Weight mean difference
