# Supplementary figures and images for: Comparison of the long-term follow-up and perioperative outcomes of partial nephrectomy and radical nephrectomy for 4 cm to 7 cm renal cell carcinoma: a systematic review and meta-analysis
Source: BMC Urol. 2019 Jun 7;19:48. doi: 10.1186/s12894-019-0480-6 (PMC6554915; doi:10.1186/s12894-019-0480-6)

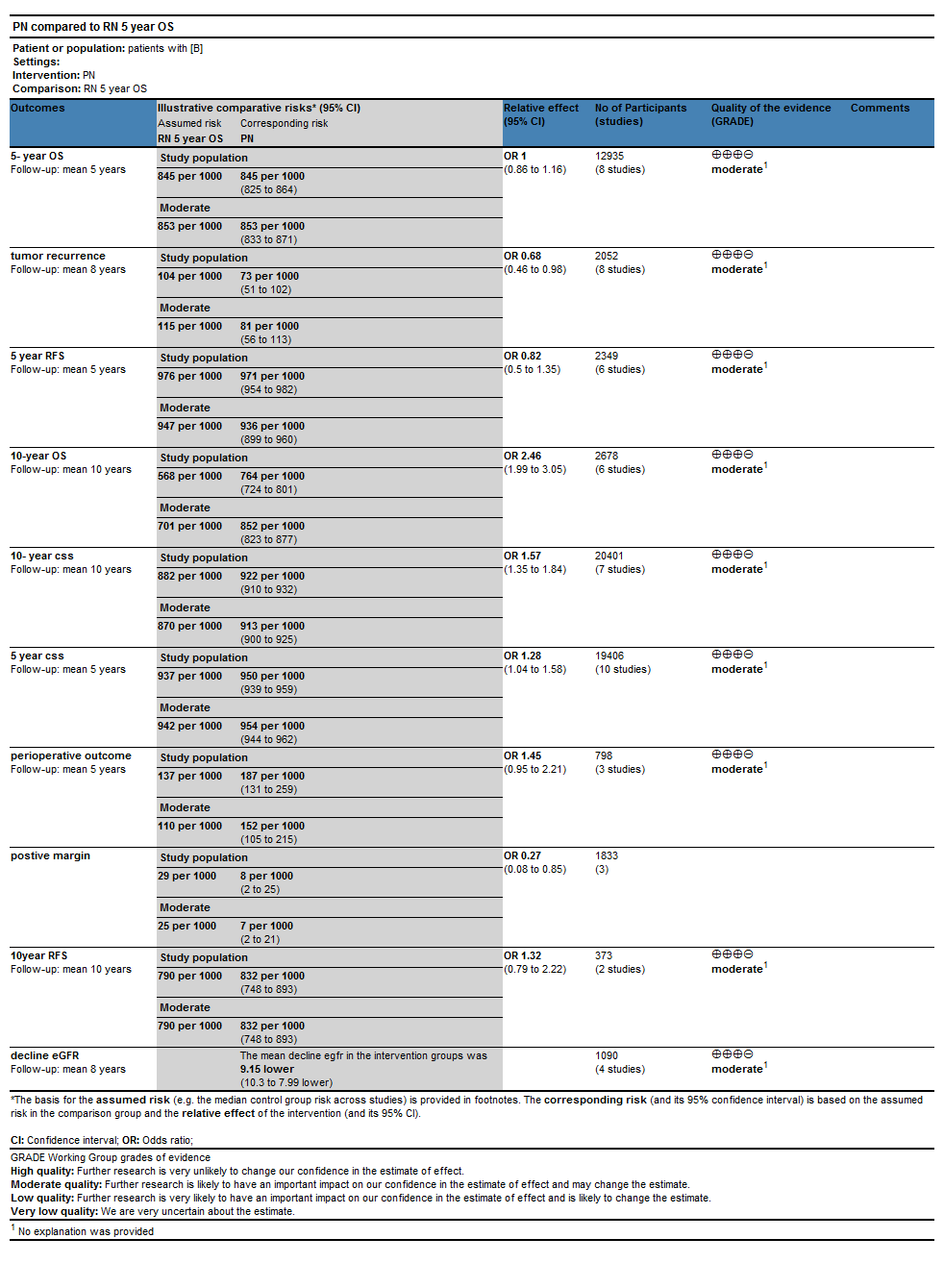

Supplement: Supplementary file 3 — Figure S3. Summary of evidence grading. (PNG 84 kb) [file 12894_2019_480_MOESM3_ESM.png]
